# Compact modulators on silicon nitride waveguide platform via micro-transfer printing of thin-film lithium niobate

**DOI:** 10.1038/s41598-025-95397-w

**Published:** 2025-04-05

**Authors:** S. Hadi Badri, Maria V. Kotlyar, Risov Das, Yeasir Arafat, Owen Moynihan, Brian Corbett, Liam O’Faolain, Samir Ghosh

**Affiliations:** 1 Centre for Advanced Photonics and Process Analysis, Munster Technological University, Cork, T12 P928 Ireland; 2 Tyndall National Institute, Lee Maltings, Dyke Parade, Cork, T12 R5CP Ireland

**Keywords:** Optics and photonics, Integrated optics

## Abstract

We explore the use of micro-transfer printing (µTP) technology to integrate thin lithium niobate (LN) films onto silicon nitride (SiN) waveguides, facilitating the development of compact electro-optical modulators. Three modulator architectures are investigated: Mach-Zehnder interferometer (MZI), Fabry-Perot (FP) resonator, and side-coupled FP resonators. By acting as a photonic molecule, the proposed coupled FP resonators enable improved spectral engineering with new functionalities while maximizing the transmission and quality-factor (Q-factor) of the resonances. Design, simulations, fabrication method, and experimental results are presented, demonstrating the potential of µTP in advancing electro-optical modulators. The half-wave voltage-length product (*V*_*π*_*L*) of the fabricated devices decreases as the Q-factor increases achieving *V*_*π*_*L =* 10.5, 4.3, and 2.74 V.cm for MZI, FP, and photonic molecule modulators, respectively.

## Introduction

The ever-growing demand for high-speed data communication is driving the development of faster and higher bandwidth data transfer technologies. Photonics offers a compelling solution, enabling not only advanced communication but also sensing, imaging, and renewable energy applications. Unlike the mature silicon electronics industry dominated by complementary metal–oxide–semiconductor (CMOS) technology, photonics lacks a single ideal material platform. Photonic integrated circuits (PICs) require a combination of properties, including a wide transparency window, efficient light emission and detection, strong electro-optic (EO) coefficient, and ease of fabrication and integration – a challenge no single material can meet. Silicon-on-insulator (SOI) is a popular platform due to its compatibility with silicon foundries. However, SOI has limitations that include the inability to create lasers, high two-photon absorption, and the lack of second-order nonlinearities. It also suffers from high absorption in the visible range and low modulator extinction ratios based on the free-carrier plasma dispersion effect. These limitations have spurred the development of alternative material platforms such as silicon nitride (SiN), doped-silicon dioxide, gallium arsenide, indium phosphide, aluminum nitride, gallium nitride, and LN^1^. Heterogeneous integration, which combines these material systems on a single substrate, offers a pragmatic solution. Various techniques like epitaxial growth^2^, bonding^3^, and µTP^4^ have been explored for this purpose. µTP stands out as a simple yet versatile micro-assembly technology with high yield. It enables fast and parallel assembly of micro-scale components under ambient conditions at lower costs as compared to the wafer bonding approach. An elastomer stamp transfers components from a source substrate to a target surface, facilitating the co-integration of dissimilar materials. µTP is particularly advantageous for integrating materials that are difficult to pattern or deposit using conventional methods. Additionally, it allows the utilization of materials that are otherwise incompatible due to processing and chemical limitations by decoupling deposition, patterning, and printing steps^4^.

In particular, today’s data centers deploy hundreds of thousands of servers across multiple hierarchical layers, necessitating an interconnection network that is not only cost-effective but also energy-efficient. New methods for the realizing of switching elements are needed that combine a high radix connectivity architecture and simultaneous bandwidth. Advanced switching architectures have proposed and demonstrated based on cyclic arrayed waveguide gratings and high speed optical modulators are key elements^5^. Thin film LN modulators are very promising for their low insertion loss and high-speed operation. LN has large Pockels effect, enabling pure phase modulation without spurious amplitude modulation – a significant advantage compared to silicon and III-V material modulators. Additionally, LN has emerged as a multifunctional platform offering a unique combination of electro-optic, acousto-optic, elasto-optic, piezoelectric, pyroelectric, and non-linear optical properties, making it a highly promising platform for active photonic devices^6^. However, LN is challenging to process, making devices fabricated with non-standard etching techniques and partially etched waveguides difficult to reproduce. To address this, various integration methods, such as wafer-scale bonding, have been explored for LN-on-SiN platforms^7,8^. µTP of a thin LN film onto a pre-patterned waveguide material such as SiN photonic integrated circuits is another attractive solution. This approach eliminates the need for precise LN etching while combining the scalability of CMOS-compatible photonics with the excellent electro-optic modulation capabilities of LN^9^. While µTP holds promise, it remains a relatively new technique with limited reported implementations in modulators. Existing demonstrations include a 2 mm-long Mach-Zehnder modulator on the LN-on-SiN platform, featuring two 1 × 2 multimode interferometers (MMIs) in a push-pull configuration. This design achieved an insertion loss of 3.3 dB and a half-wave voltage (*V*_*π*_) of 14.8 V^10^. Another study reported a ring modulator fabricated by micro-transfer printing a 600 nm-thick LN film on silicon, exhibiting an insertion loss of 1.5 dB and an extinction ratio of 37 dB^11^. A mid-infrared modulator has been demonstrated using micro-transfer printed silicon on LN. This modulator achieves a half-wave voltage length product of 12.3 V∙cm at a wavelength of 3.78 μm and exhibits a maximum extinction ratio of 25.2 dB^12^. SiN loaded and direct patterned LN cross-sections have also been investigated for achieving improved second harmonic generation through engineered lateral leakage^13^. These works highlight that LN on insulator is emerging as a viable PIC platform.

In this paper, we explore the implementation of electro-optic modulators using µTP of thin LN films onto a patterned SiN target. To realize efficient modulators, long device lengths are currently required^14^. Reduced footprints are essential, particularly if the field is to advance to switches with even higher port counts. Resonant enhancement is an important principle to enhance light matter interaction and realize high performance with short device lengths^15^. The low refractive index contrast LN-on-SiN imposes challenges on bending radius, making Fabry-Perot (FP) microcavities a very promising route^16^. To this end, we demonstrate modulators based on three different architectures: MZI, in-line FP resonator, and side-coupled FP resonators. An in-line FP cavity consists of a resonant region enclosed by two mirrors. Increasing the Q-factor requires higher mirror reflectivity to trap light for longer durations. Minimizing cavity losses can further enhance performance without significantly compromising transmission but, scattering loss tends to be constant for a given fabrication process. Therefore, a fundamental trade-off exists in FP resonators: in the presence of loss, maximizing the Q-factor reduces the transmission peaks, and vice versa. To the best of our knowledge, this is the first demonstration of a modulator based on a side-coupled FP resonators, a novel photonic molecule architecture with properties analogous to molecules, to maximize the transmission-Q-factor product. This photonic molecule leverages controlled mode interaction between the coupled resonators, enabling us to simultaneously achieve high Q-factor and increased light transmission, offering superior flexibility for spectral engineering compared to conventional FP resonators. The photonic molecules have found interesting applications such as comb generation^17^, quantum optics^18^, lasing^19^, and sensing^20^.

## Design and simulations

This section details the design considerations and simulations performed for the electro-optic modulators fabricated using micro-transfer printed LN films on SiN waveguides. We analyze the optical transmission characteristics, electro-optic effect, and performance of the aforementioned modulators.

**Fig. 1:**
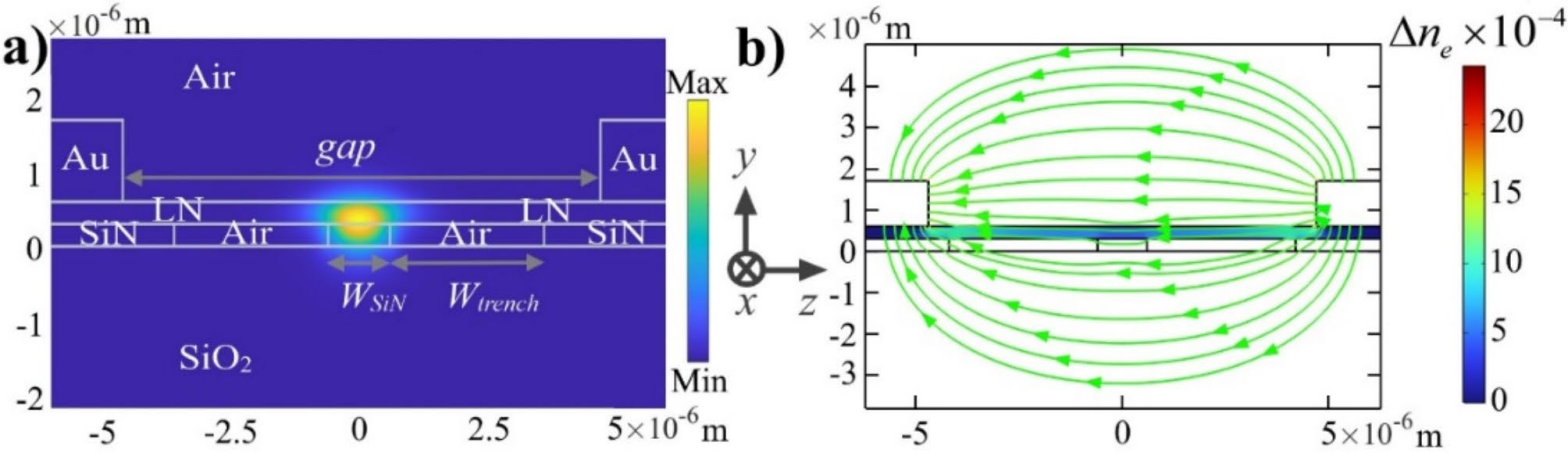
(**a**) Simulated TE mode intensity in the hybrid LN-on-SiN waveguide at 1550 nm. (**b**) Electric field distribution and resulting refractive index change in the LN layer with 40 V applied to the electrodes and a gap of *gap =* 8.2 μm.

The optical field distribution of the transverse electric (TE) mode in the LN-on-SiN waveguide at the wavelength of 1550 nm is shown in Fig. 1a. The design of the LN-on-SiN waveguide geometry was guided by both fabrication constraints and numerical simulations. The thickness of the SiN, LN, and electrode layers was determined by material availability and fabrication feasibility. Simulations using Lumerical MODE Solutions indicate that reducing the SiN width increases mode confinement in the LN slab but also causes greater lateral mode expansion. This necessitates a larger electrode gap, which in turn weakens the EO effect. For example, at *W*_*SiN*_ = 0.6 μm, the mode confinement in LN increases to 65%, whereas at *W*_*SiN*_ = 1.4 μm, it decreases to 55%. To balance confinement and EO efficiency, we selected a SiN strip waveguide width of *W*_*SiN*_ = 1.2 μm, while the trenches on either side have a width of *W*_*trench*_ = 3 μm. Both the SiN layer and the *x*-cut LN film have a thickness of 300 nm. These waveguide parameters result in an optical field confinement of about 57% in LN slab as shown in Fig. 1a. The effective index of the mode is 1.82 while the group index is 2.19 at 1550 nm. LN exhibits a linear electro-optic effect (Pockels effect), making it an ideal material for optical modulator. To achieve efficient modulation, it is crucial to align the applied electric field along the extraordinary axis of the LN film. The 1.1 μm-thick gold (Au) electrodes are placed on both sides of the waveguide. While reducing the gap would enhance the EO interaction, precise alignment of electrodes at very small gaps poses fabrication challenges. Potential misalignment or variations in electrode positioning could lead to asymmetric field distribution, reduced modulation efficiency, or increased optical loss. To ensure reliable device performance across multiple fabrication runs, we adopted a conservative approach choosing varying electrode gaps. Figure 1b illustrates the electric field distribution and the resulting change in the LN’s refractive index. Figure 1b shows that, the *E*_*z*_ component of the electric field dominates, while *E*_*y*_ component is negligible and *E*_*x*_=0. Therefore, the perturbed refractive index distribution can be simplified as^21,22^

1 $$\Delta {n_e}(y,z)= - n_{e}^{3}{r_{33}}{E_z}/2$$

where the extraordinary refractive index of LN is *n*_*e*_ = 2.14 while its linear electro-optic coefficient is *r*_*33*_ *=* 30.9 pm/V at the wavelength of 1550 nm^23^. This linear relationship enables precise control over the refractive index by tuning the applied electric field. Consequently, both positive and negative refractive index changes can be achieved by controlling the electric field direction.

**Fig. 2:**
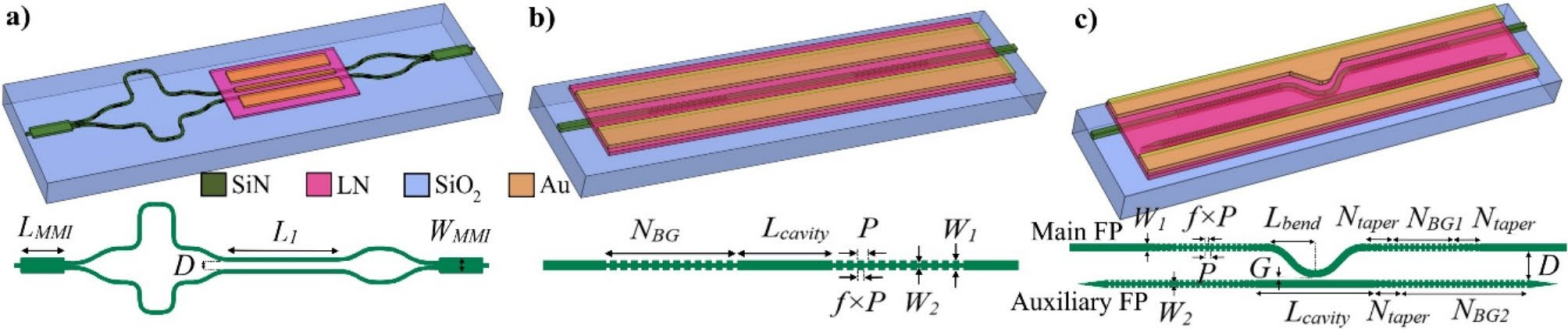
Schematic of the designed modulators and their corresponding patterned SiN layers based on (**a**) Mach-Zehnder interferometer (MZI), (**b**) Fabry-Pérot (FP), and (**c**) side-coupled FP resonators as a photonic molecule.

The schematic of the designed modulators based on MZI, FP resonator, and photonic molecule architectures are presented in Fig. 2 with the detailed design descriptions provided in the following subsections.

### MZI modulator

A schematic of the Mach-Zehnder interferometer modulator (MZM) is shown in Fig. 2a. A multimode interference (MMI) device splits the light equally into the two arms of the MZM, which have different optical path lengths. A second MMI device combines the light from the arms into the output waveguide. The MMI is 33.2 μm long (*L*_*MMI*_) and 4.5 μm wide (*W*_*MMI*_). The MZM has a length imbalance of 100 μm between the arms which gives a free-spectral-range (FSR) of 12.5 nm at the wavelength of 1550 nm. This facilitates measuring EO-effect induced spectral shifts. The arms are strategically routed so that the ground-signal-ground (G-S-G) electrodes can be fitted along the MZM arms without crossing the optical waveguide. The length of the straight section of the arms (*L*_*1*_) is 2.4 mm where the LN coupon was directly printed using transfer print with the assistance of suitable alignment marks. The spacing between the arms (*D*) is 27.25 μm which leaves a sufficient room to place the 23.5 μm wide signal electrode. Ground pads are placed on the outer sides of the respective arms. The gap between the signal and ground electrodes is 9.2 μm.

### FP modulator

FP resonators are fundamental elements in photonics, finding applications in diverse areas^24,25^. They are essentially cavities enclosed by highly reflective mirrors, often implemented as distributed Bragg reflector (DBR). In our previous work, we have realized LN-FP microcavities based on strip loaded waveguides, in which a silicon nitride layer was deposited on the LN layer using plasma enhanced chemical vapor deposition (PECVD)^16^. Here, we adapt and optimize this design for the micro-transfer printed LN approach, which has important advantages in that the silicon nitride fabrication is completed first. Moreover, high performance, low loss components can be realized without restrictions on the thermal budget, or the limitations of PECVD imposed in our previous work^16^.

We designed a Fabry Perot micro-cavity modulator that is defined by two DBRs separated by a waveguide. The DBR is realized via a periodic modulation of the width of the SiN sections as shown in Fig. 2b, which creates a modulation of the effective refractive index of the optical mode. The geometrical parameters of the FP resonator are *P =* 440 nm, *W*_*1*_ = 1200 nm, *W*_*2*_ = 800 nm, *f =* 0.6, *N*_*BG*_ = 150, and *L*_*cavity*_ = 660 μm. A corrugation width of 400 nm was chosen to maximize the bandwidth and reflectivity of the DBR within the fabrication constraints.

The number of periods in the DBR was chosen to *N*_*BG*_ = 150 periods as this provides a trade-off between Q-factor and transmission peak of the resonances. Increasing *N*_*BG*_, leads to higher Q-factor, however, coupling to the cavity decreases exponentially with increasing *N*_*BG*_, causing a rapid decrease in transmission. Achieving both high Q-factor and high transmission level at the same time is not possible, see^16^ for a detailed consideration of the effects of the number of periods on device performance. Based on our simulations, *N*_*BG*_ = 150 was the most satisfactory compromise as it maximized the product of Q-factor and transmission.

The DBR-FP provides ease of fabrication and control over the FSR and Q-factor. It is well known that the modulation bandwidth is restricted by the photon lifetime in any optical resonator. By changing the cavity length, the optimum trade-off between modulation extinction ratio (which is influenced by Q-factor) and bandwidth (inversely proportional to Q-factor) can be selected for a particular application.

### Side-coupled FP modulator

When two resonators are placed in close proximity, the evanescent field of one resonator can perturb the field of the other. This coupling of resonators splits the original resonance frequency into a doublet of two new eigenfrequencies, a characteristic of the coupled system. These new frequencies correspond to the modes of the coupled system, often referred to as supermodes. Such coupled resonators are often called photonic molecules since the optical modes in them are similar to the electronic states of diatomic molecules^26–28^.

**Fig. 3:**
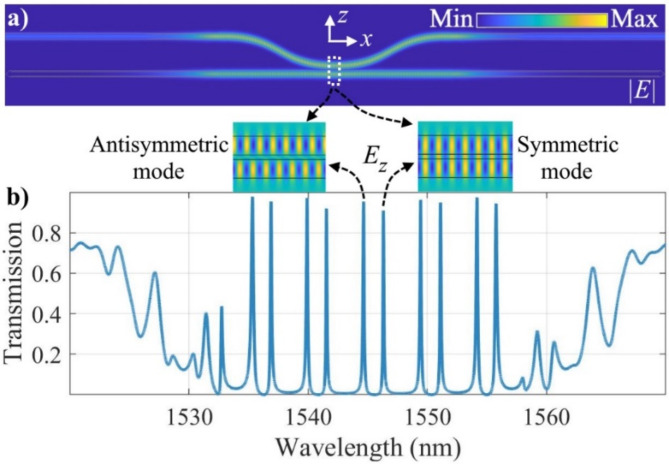
(**a**) Intensity of the optical field at the resonance wavelength of 1544.7 nm. The *E*_*z*_ component of the optical field in the coupling region is also shown. (**b**) Simulated transmission spectrum of the side-coupled FP resonators.

Here, we present a photonic molecule, i.e., side-coupled FP resonators, to manipulate the mode interaction, resulting in the splitting of the original resonance into two new supermode resonances, including a shorter-wavelength antisymmetric and longer-wavelength symmetric optical modes. In this design, both the Q-factor as well as the transmission peaks of the resonances can be increased simultaneously - a significant advantage compared to simple FP resonators, for example those discussed in the previous section. The proposed structure consists of two FP resonators, namely, the main and auxiliary FP resonators evanescently coupled to each other as shown in Fig. 2c. Each FP resonator is composed of a cavity and two DBRs on either side of the cavity. We taper the DBRs to transition from the waveguide mode to Bloch mode and vice versa, considerably reducing the DBR-waveguide coupling loss. The sharp tips at the end of the DBRs in the auxiliary FP eliminate the back reflection. To model this advanced design, we employed Lumerical’s VarFDTD tool to simulate the proposed structure. Perfectly matched layer (PML) boundary conditions and non-uniform mesh accuracy of 3 settings were applied in the Lumerical simulation. The optical field distribution in the photonic molecule is shown in Fig. 3a at the resonance wavelength of 1544.7 nm. The shorter-wavelength antisymmetric and longer-wavelength symmetric optical modes at the coupling region of the photonic molecule are also shown. The transmission spectrum of the structure is shown in Fig. 3b. The geometrical parameters used in the presented simulation results are *P* = 430 nm, *f* = 0.6, *W*_*1*_ = 1200 nm, *W*_*2*_ = 700 nm, *G* = 300 nm, *L*_*bend*_ = 50 μm, *L*_*cavity*_ = 104.92 μm, *D =* 7 μm, *N*_*BG1*_ = 50, *N*_*BG2*_ = 200, and *N*_*taper*_ = 20. The length of the cavity is the same for both the cavities.

**Fig. 4:**
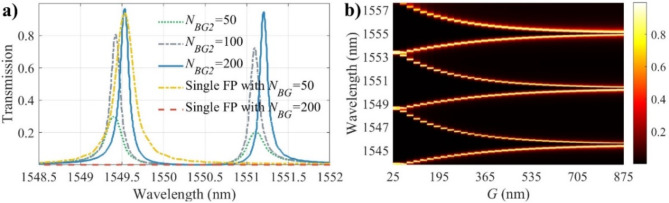
(**a**) Simulated transmission spectra vs. the number of gratings in the auxiliary FP. Increasing the DBR reflectivity in the auxiliary FP leads to higher Q-factor and transmission level. Also the performance of the photonic molecule is compared with a single FP. (**b**) Simulated tansmission spectra vs. the gap between the FP resonators. Decreasing the gap leads to higher coupling strength pushing the split resonances away from each other.

As shown in Fig. 4a, increasing the number of gratings in the auxiliary resonator from *N*_*BG2*_ = 50, 100, to 200 leads to a significant improvement in Q-factor, reaching values of 6200, 11,900, and 14,100 at the wavelength of 1551.1 nm, respectively. Simultaneously, the transmission peak also increases from 0.20, 0.72, to 0.96, respectively. Notably, increasing the DBR reflectivity in the auxiliary FP leads to a divergence in the Q-factors of the split resonances. For instance, Q-factors of 12,880 and 14,440 are achieved for *N*_*BG2*_ = 200 at the resonance wavelengths of 1549.42 and 1551.10 nm, respectively. The Q-factor of a conventional FP with *N*_*BG*_ = 50 is 6,710. On the other hand, no resonances are observed in a single FP with *N*_*BG*_ = 200 in the middle of the bandgap, as the increased DBR reflectivity minimizes the light penetration into the cavity. However, close to the bandgap edge–where the DBR reflectivity is lower compared to the center–there is a resonance (not shown in Fig. 4a) with a transmission peak of 0.16 and a Q-factor of 618,000. Although the Q-factor of this resonance is higher than that achieved with the photonic molecule, its transmission level is significantly lower. The lower Q-factor of the photonic molecule arises from losses introduced by the bent waveguide and coupling between the FP resonators. Overall, these results highlight that the Q-factor in the coupled FP resonators can be increased while allowing for higher transmission levels at the resonances. The splitting strength is proportional to the inter-FP coupling strength which depends on the gap between the resonators and the coupling length. This behavior is analogous to that observed in coupled ring resonators^29^. As shown in Fig. 4b, by changing the inter-FP coupling strength, in this case, the gap between the resonators (*G*), the split resonances can be pulled towards each other or pushed further away. For instance, by increasing the gap from *G =* 150, 300, to 450 nm, the split between the resonances decreases from 2.97, 1.67, to 0.96 nm, respectively. Obviously, by further increasing the gap between the FPs, the splitting decreases further and ultimately, we would have two uncoupled FPs.

## Fabrication

The designed LN-on-SiN modulators contain a thin-film LN as an EO material printed on top of the patterned SiN PIC. We utilize micro-transfer printing to pick the LN coupon up from the Lithium niobate-on-insulator (LNOI) source chip and print it onto the desired location on the SiN target chip. Gold electrodes are deposited on both sides of the structure to apply an electric field across the optical waveguide, modulating the light propagation characteristics.

First, we describe the fabrication procedure of the target chip, i.e., waveguide patterning on SiN. The fabrication process begins with a commercially available SiN wafer from LioniX, featuring a 300 nm thick low-pressure chemical vapor deposition (LPCVD) SiN layer on a 2 μm buried oxide (BOX) grown on a silicon substrate. We utilize electron beam lithography (EBL) with a 100 kV Elionix Ebeam exposure tool to define the desired waveguide patterns on the SiN layer. After exposure, the photoresist is developed to create a patterned resist mask. An inductively coupled plasma (ICP) etch process with a mixture of CHF_3_ and O_2_ gases is used to transfer the resist mask pattern into the underlying SiN layer. Following the waveguide etch, a second EBL step defines the grating coupler (GC) patterns on both ends of the waveguides. GCs facilitate efficient vertical light coupling between the on-chip waveguides and standard single-mode fibers (SMFs). Unlike the fully etched waveguides, the GCs are only partially etched to maintain a defined grating profile for efficient light coupling. Another ICP etch process similar to the waveguide etch is performed to create the grating structures on the SiN layer, defining the GCs. Here, the etch depth is precisely controlled to achieve the desired 150 nm etch depth for optimized light coupling efficiency.

**Fig. 5:**
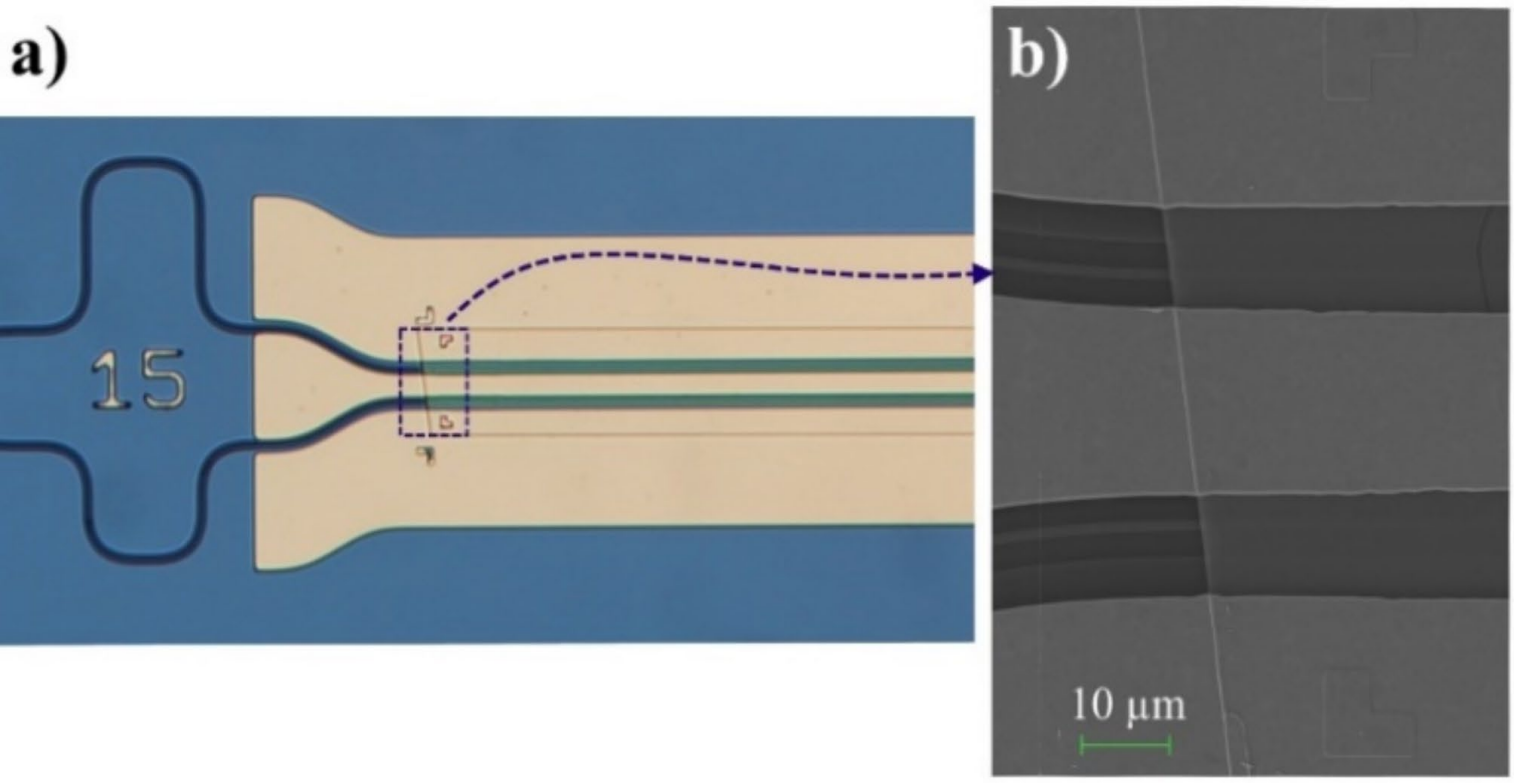
(**a**) Optical image of the MZI modulator. (**b**) SEM image showing the transition where LN meets SiN waveguide.

LN coupons, measuring 2.4 mm in length and 100 μm in width, are prepared from commercially available *x*-cut LNOI wafer containing 300 nm-thick LN layer on top of 2 μm thick BOX grown on silicon substrate. The coupon boundary is first defined by optical lithography followed by ICP etching into the silicon substrate. A second optical lithography step is performed to define the tethering structure, which consists of resist features that temporarily hold the coupons in place during the undercut process. The BOX layer is then released by undercutting in buffered oxide etchant (BOE). The integration of LN onto SiN waveguide was accomplished in µTP tool. To achieve adequate print adhesion between LN and SiN, SiN target was treated in O_2_ plasma for 5 minutes prior to the printing. A polydimethylsiloxane (PDMS) stamp, with a size slightly bigger than the coupon, is used to pick the LN coupon —by breaking the tethering structure— and place it onto the target SiN chip with the desired alignment. Any remaining tether resist was removed by solvent, ensuring that the devices are clean and ready for electrode deposition. The final step involves depositing metal electrodes on both sides of the LN-on-SiN structure, which are patterned using lift-off lithography. These electrodes allow us to apply an electric field across the LN layer, inducing a change in its refractive index and modulating the light propagation characteristics within the waveguides. Figure 5a presents an optical image of the completed MZI modulator device. Scanning electron microscope (SEM) image in Fig. 5b is showing the transition where LN meets SiN waveguide. The alignment of the LN coupon relative to the SiN structure is not highly restrictive due to the coupon’s width (100 μm) being significantly larger than the underlying SiN features. As a result, the 2 μm accuracy of our µTP process has no significant impact on device performance. However, angular alignment is more critical since LN is an anisotropic material, meaning even slight misalignment can influence the electro-optic response. In our process, angular misalignment is kept below 0.2°, which remains within acceptable limits for structural placement. Nevertheless, further improving angular precision could enhance modulation efficiency. The electrodes were patterned using lift-off lithography with an accuracy of approximately 1 μm. It is important to note that the abrupt transition from the SiN waveguide to the SiN-on-LN waveguide introduces optical loss due to mode mismatch between the two waveguides. This loss could be minimized by incorporating a tapered transition, which would gradually adapt the mode profile and improve coupling efficiency.

## Measurements and results

**Fig. 6:**
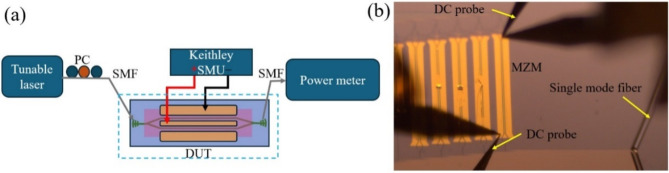
(**a**) The schematic of the measurement setup. (**b**) The microscopic image of a device during active measurement. Labels: *PC* polarization controller, *SMF* single mode fiber, *DUT* device under test, *SMU* source-measure unit, *DC* direct current, *MZM* Mach-Zehnder interferometer modulator.

This section details the characterization of the fabricated devices: the MZI modulator, the FP modulator, and the side-coupled FP modulator. The GCs on either side of the devices vertically couple the light to the single mode fibers (SMF). As the GCs are highly polarization-dependent and designed for TE polarization, we use a polarization controller (PC) to ensure TE mode excitation within the devices. A tunable laser source sweeps across the desired wavelength range, while the output light from the other end is collected and measured by a power meter. The transmission measurements were normalized to the transmission of a SiN waveguide without an LN layer to account for losses introduced at the SiN-LN interface. The interface loss was determined by comparing the transmission of a bare SiN waveguide before transfer printing to that of an LN-on-SiN waveguide, yielding an estimated loss of ~ 5 dB. This interface loss significantly contributes to the overall insertion loss of the modulators and must be considered when analyzing their performance. A Keithley source-measure unit (SMU) applies voltage to the electrodes via standard direct current (DC) probes. The probe tip is narrower than the electrodes for precise contact. Schematic in Fig. 6a depicts the setup used to measure DC phase shift of fabricated devices while Fig. 6b shows the optical microscopic image of a probed device (MZM) during active measurement.

### MZI modulator

**Fig. 7:**
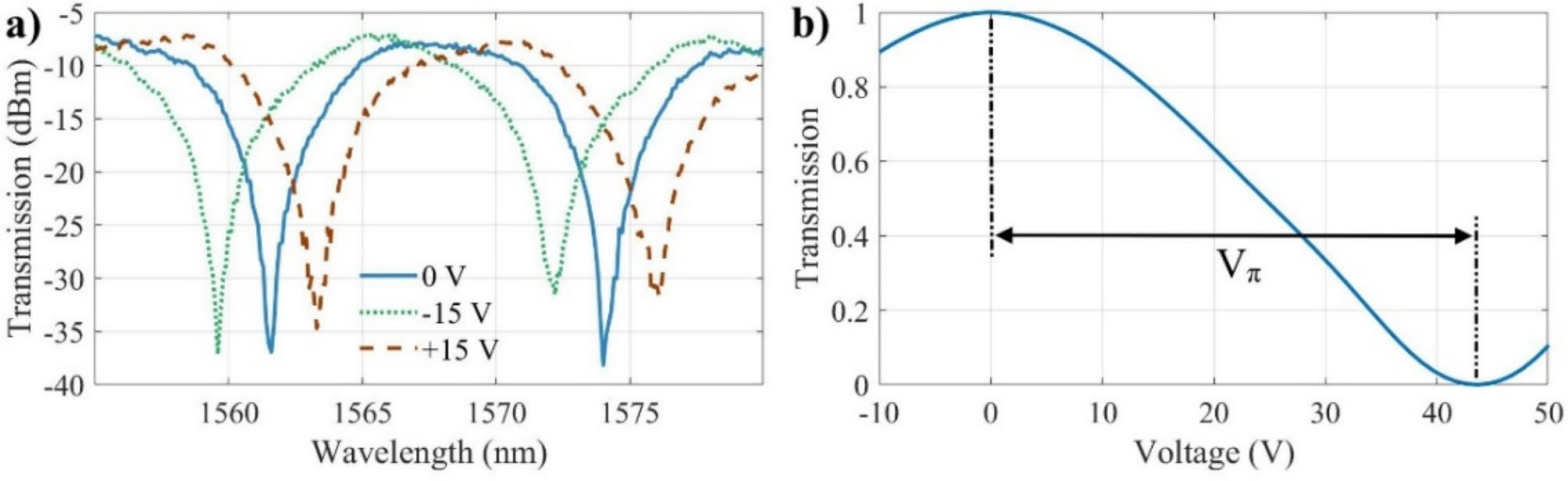
(**a**) Measured spectra of MZI modulator under different voltages. (**b**) Normalized output power as a function of applied DC voltage at 1550 nm.

Figure 7a shows the measured transmission spectra of the MZI modulator with a length of 2.4 mm under various applied voltages. The gap between the electrodes is 9.2 μm. The measured FSR is 12.5 nm at the wavelength of 1550 nm. The MZI modulator demonstrates an insertion loss of less than 8 dB and an extinction ratio of approximately 27 dB. The high extinction ratio of the MZI modulator can be attributed to its significant arm separation, which enhances the interference contrast by minimizing unintentional coupling between the arms. This separation reduces phase noise and fabrication-induced asymmetries, leading to improved extinction performance. The measured wavelength shifts are approximately 0.6, 1.2, 1.8, and 2.4 nm for applied voltage of 5, 10, 15, and 20 V, respectively. For readability, Fig. 7a only shows the measured transmission spectra for ± 15 V. The observed modulation behavior under both positive and negative applied voltages confirms that the device operates based on the linear EO (Pockels) effect rather than thermo-optic effects. Unlike thermo-optic modulation, which is generally slow and unidirectional, the electro-optic effect is instantaneous and symmetric, meaning that reversing the applied electric field results in an opposite phase shift. The measured response of our devices to both positive and negative voltages strongly supports this electro-optic nature. Figure 7b shows the normalized output power as a function of applied DC voltage measured at the wavelength of 1550 nm. *V*_*π*_, the voltage required to induce a π phase shift in the light, is measured to be 43.6 V for single phase shifter. *V*_*π*_.*L* of this device under push-pull operation is 10.5 V.cm.

### FP modulator

**Fig. 8:**
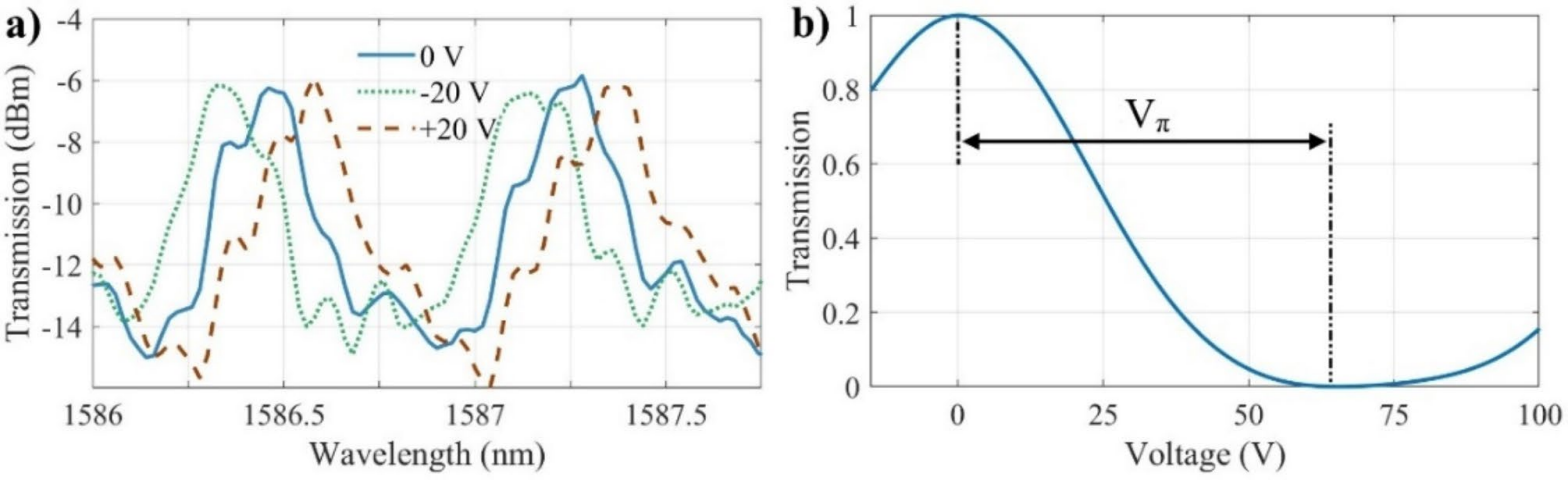
(**a**) Measured spectra of FP modulator under different voltages. (**b**) Normalized output power as a function of applied DC voltage at 1550 nm.

The measurement results for the FP modulator, with a cavity length of 660 μm, are presented in Fig. 8a. The gap between the electrodes is 15.0 μm. Resonance wavelength shifts of 40, 80, and 120 pm are recorded when the applied voltage changes from 10, 20, to 30 V, respectively. We only illustrate the measured transmission spectra for ± 20 V in Fig. 8a. The FSR is approximately 0.8 nm, with an insertion loss of less than 7 dB and an extinction ratio of around 8.5 dB. As shown in Fig. 8b, the measured *V*_*π*_ measured at the wavelength of 1550 nm for this device is 65.2 V with *V*_*π*_*L* product as 4.3 V.cm. During the experimental characterization, unwanted fluctuations were observed in the measured spectra of the devices under different applied voltages. These fluctuations are seen in the same device for different voltages, indicating that they are unlikely to originate from intrinsic device effects. Instead, we attribute them primarily to measurement noise.

### Side-coupled FP modulator

**Fig. 9:**
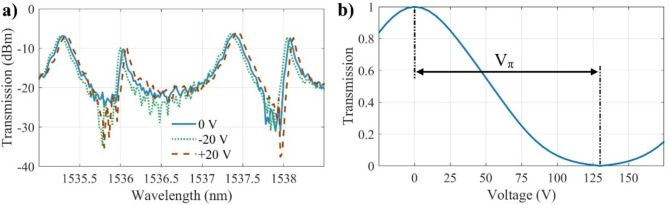
(**a**) Measured spectra of side-coupled FP modulator under different voltages. (**b**) Normalized output power as a function of applied DC voltage at 1550 nm.

Figure 9a presents the measured transmission spectra of the side-coupled FP modulator with *L*_*cavity*_ = 209.84 μm, *G =* 600 nm, *N*_*BG1*_ *=* 100, *N*_*BG2*_ *=* 1000, and *L*_*bend*_ *=* 100 μm under various applied voltages. The other geometrical parameters are identical to those described in the Sect. 2.3. In the fabricated devices, we used a longer *L*_*bend*​_ and *L*_*cavity*_, as well as stronger mirrors with a larger number of Bragg gratings, to enhance performance. However, in our simulations, we employed smaller values for these parameters to manage computational cost while maintaining accurate representations of the device physics. The gap between the electrodes is 15.2 μm (note that this is larger than the gap used in Sect. 4.1). Approximately, 0.7 nm splitting is demonstrated in this photonic molecule. The insertion loss is less than 7 dB while the extinction ratio is about 15 dB. The measured *V*_*π*_ measured at the wavelength of 1550 nm is 130.2 V for this device with *V*_*π*_*L* as 2.74 V.cm as shown in Fig. 9b. The resonance wavelength shifts by 40 and 80 pm when the applied voltage is 20 and 40 V, respectively. Only the measured transmission spectra for ± 20 V are illustrated in Fig. 9a. The measured Q-factors of the split resonances are 19,500 and 52,200. This significant divergence in Q-factors arises from the substantial difference in the reflectivity of the DBRs within the two FP resonators.

## Discussion

As presented above, three different LN-SiN hybrid electro-optic modulator architectures are designed, and their performance compared experimentally. A route towards the realization of compact devices is shown. High-speed data transmission experiments cannot be performed with these devices due to high *V*_*π*_, a consequence of the large electrode gap. The obvious way to lower the *V*_*π*_ is to increase the EO interaction. This can be achieved by shrinking the electrode gap further and increasing the mode confinement into the LN layer. While this strategy would improve the EO interaction, several important design trade-offs need to be addressed. Increasing mode confinement in the LN layer results in a larger mode mismatch at the interface between the SiN and LN-on-SiN waveguides, making it essential to use tapers to minimize reflections. Furthermore, it causes lateral mode expansion in the LN layer, necessitating a larger electrode gap that weakens the EO effect. Conversely, reduced field overlap with the SiN gratings can lower the reflectivity of the DBRs, potentially requiring longer DBRs to compensate for this effect. Furthermore, although shrinking the electrode gap strengthens the applied electric field, it also increases the overlap between the optical mode and the lossy metal electrodes, leading to higher optical absorption. Striking an optimal balance between electrode placement and mode confinement is crucial to maximize modulation efficiency while minimizing loss.

In this work, unpatterned LN is transferred onto SiN waveguide which eases the integration process since no alignment between LN and SiN is required. However, patterning LN will be necessary to lower the *V*_*π*_*L* product. LN can be patterned after the transfer which will require a strategy to protect exposed SiN waveguide from being damaged by the plasma during LN etching. Alternatively, patterned LN can be transferred onto SiN waveguide which will require strict alignment accuracy between SiN and LN waveguides. A potential drawback of this approach is that the patterned LN waveguide might give increased waveguide loss due to sidewall roughness. Therefore, waveguide dimensions and fabrication process will be important considerations. There is also further room to improve the SiN waveguide loss by optimizing EBL process and ICP etching recipe. The reliability of printed coupons is also a crucial factor for actual applications and studies of the yield will be performed in future works. It is known that any liquid like solvent or deionized water can flow through the waveguide trench beneath the LN coupon. If that liquid cannot escape, then it may lead to coupon debonding. Therefore, a suitable strategy is required to block the trench where SiN/LN interface will need to be developed. The power consumption of the MZM is estimated based on measured current values. At an applied voltage of 20 V, the measured current was 5.1 nA, resulting in a power consumption of approximately 102 nW. Table 1 summarizes the performance metrics of various integrated heterogenous LN modulators, highlighting the achieved insertion loss, extinction ratio, *V*_*π*_*L*, and bandwidth. The data underscores the potential of µTP to create compact modulators with diverse architectures, paving the way for advanced photonic integrated circuits. While our current devices prioritize footprint reduction and integration flexibility, future work will focus on reducing the electrode gap (which was chosen conservatively in this work) and optimizing RF design to further improve high-speed performance. Additionally, the photonic molecule architecture presented in this work is particularly promising, as it achieves a low *V*_*π*_*L* while enabling spectral engineering.

**Table 1:** Comparison of heterogeneous LN modulators.

Modulator type	Material platform	Integration method	Insertion loss (dB)	Extinction ratio (dB)	VπL (V cm)	Bandwidth (GHz)	References
MZM	LN-on-Si	die-to-wafer bonding	1.8	28	3.1	110	^30^
MZM	LN-on-SiN	µTP	3.3	39	2.9	> 50	^10^
MZM	LN-on-SiN	die-to-die bonding	1	> 30	3.0	37	^31^
Ring resonator	LN-on-Si	µTP	1.5	37	7.0	16	^11^
MZM	LN-on-SiN	µTP	8	27	10.5	N/A	This work
FP	LN-on-SiN	µTP	7	8.5	4.3	N/A	This work
Photonic molecule	LN-on-SiN	µTP	7	15	2.74	N/A	This work

## Conclusion

In conclusion, this work demonstrated the development of compact electro-optical modulators through µTP of thin LN films onto patterned SiN substrates. We explored three modulator architectures: MZI, FP resonator, and a novel side-coupled FP design inspired by photonic molecule principles. The side-coupled FP architecture offers a compelling solution to the trade-off between high Q-factor resonances and light transmission in conventional FPs, enabling advanced spectral engineering functionalities. Notably, the fabricated side-coupled FP modulator achieved a *V*_*π*_*L* of 2.74 V.cm compared to 10.5 V.cm and 4.3 V.cm for MZI and FP designs, respectively. By leveraging the complementary strengths of SiN’s well-established fabrication and LN’s superior electro-optic properties, this µTP-based approach presents a promising pathway towards high-performance, miniaturized modulators.

## Data Availability

The datasets used and/or analyzed during the current study available from the corresponding author on reasonable request.
